# Pyrrolidinium chloride

**DOI:** 10.1107/S1600536809006060

**Published:** 2009-02-25

**Authors:** Helene Giglmeier, Tobias Kerscher, Peter Klüfers, Peter Mayer

**Affiliations:** a Ludwig-Maximilians Universität, Department Chemie und Biochemie, Butenandtstrasse 5–13 (Haus D), 81377 München, Germany

## Abstract

The title compound, C_4_H_10_N^+^·Cl^−^, was obtained as a decomposition product from 2,6-bis­(pyrrolidin­yl)pyridine. The anion lies on the same cristallographic mirror plane as the N atom of the cation, the complete cation being generated by mirror symmetry. The anions and cations are connected by N^+^—H⋯Cl^−^ hydrogen bonds into chains along [100]. The pyrrolidinium cation is puckered in an envelope conformation *E*_N1_.

## Related literature

For details of the synthesis of 2,6-bis­(pyrrolidin­yl)pyridine, see: Folmer-Anderson *et al.* (2005 ▶). For related structures containing the pyrrolidinium cation, see: Kashino *et al.* (1978 ▶); Moritani *et al.* (1987 ▶); Jakubas *et al.* (2005 ▶). For a description of the *E*_N1_ conformation of the five-membered ring, see: Cremer & Pople (1975 ▶).
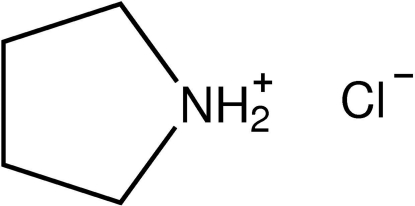


## Experimental

### 

#### Crystal data

C_4_H_10_N^+^·Cl^−^
*M* *_r_* = 107.58
Orthorhombic, 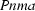

*a* = 7.4429 (4) Å
*b* = 9.4104 (5) Å
*c* = 8.9021 (4) Å
*V* = 623.51 (5) Å^3^
*Z* = 4
Mo *K*α radiation
μ = 0.48 mm^−1^
*T* = 200 K
0.22 × 0.13 × 0.12 mm

#### Data collection

Nonius KappaCCD diffractometer
Absorption correction: none
4239 measured reflections
756 independent reflections
608 reflections with *I* > 2σ(*I*)
*R*_int_ = 0.037

#### Refinement

*R*[*F* ^2^ > 2σ(*F* ^2^)] = 0.040
*wR*(*F* ^2^) = 0.120
*S* = 1.07
756 reflections
31 parameters
H-atom parameters constrained
Δρ_max_ = 0.21 e Å^−3^
Δρ_min_ = −0.24 e Å^−3^

### 

Data collection: *COLLECT* (Nonius, 2004 ▶); cell refinement: *SCALEPACK* (Otwinowski & Minor, 1997 ▶); data reduction: *DENZO* (Otwinowski & Minor, 1997 ▶) and *SCALEPACK*; program(s) used to solve structure: *SHELXS97* (Sheldrick, 2008 ▶); program(s) used to refine structure: *SHELXL97* (Sheldrick, 2008 ▶); molecular graphics: *ORTEP-3* (Farrugia, 1997 ▶); software used to prepare material for publication: *SHELXL97*.

## Supplementary Material

Crystal structure: contains datablocks I, global. DOI: 10.1107/S1600536809006060/bi2348sup1.cif

Structure factors: contains datablocks I. DOI: 10.1107/S1600536809006060/bi2348Isup2.hkl

Additional supplementary materials:  crystallographic information; 3D view; checkCIF report

## Figures and Tables

**Table 1:** Hydrogen-bond geometry (Å, °)

*D*—H⋯*A*	*D*—H	H⋯*A*	*D*⋯*A*	*D*—H⋯*A*
N1—H101⋯Cl1	0.92	2.17	3.091 (3)	180
N1—H102⋯Cl1^i^	0.92	2.18	3.097 (2)	177

Symmetry code: (i) 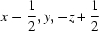.

**Fig. 1.**
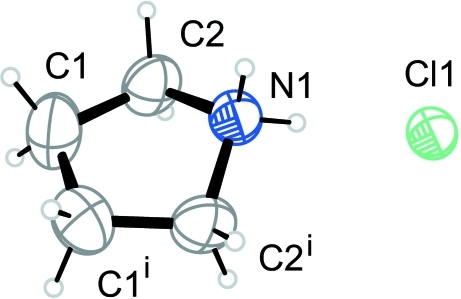
The molecular structure of the Cs symmetric title compound with anisotropic displacement ellipsoids drawn at 50% probability for non-H atoms. Symmetry code: (i) x, -y + 1/2, z.

**Fig. 2.**
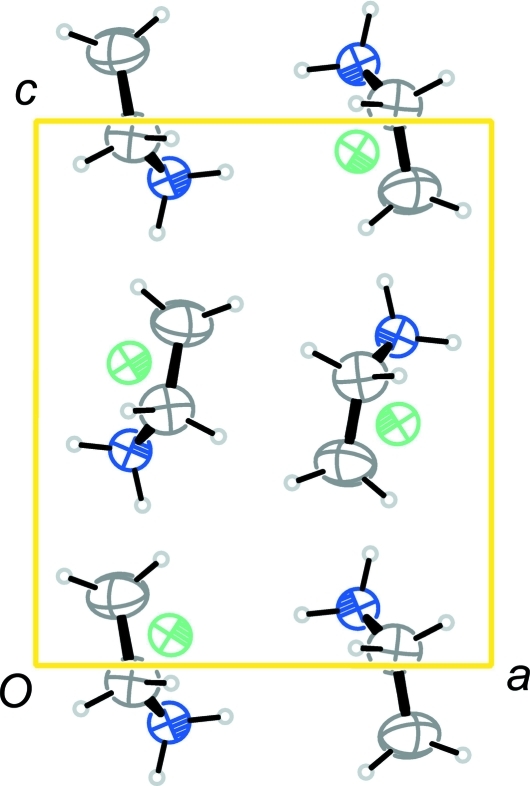
Packing of the title compound, viewed along [0 1 0].

**Fig. 3.**
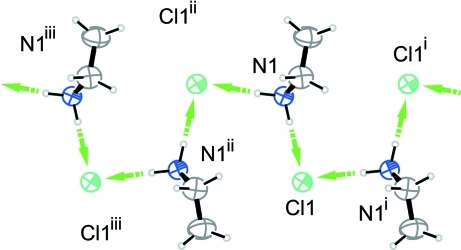
N—H···Cl hydrogen bonds lead to chain-like structures in the crystal structure along [1 0 0], shown here normal to [0 1 0]. Symmetry codes: (i) x + 1/2, -y + 1/2, -z + 1/2; (ii) x - 1/2, -y + 1/2, -z + 1/2; (iii) x - 1, y, z.

## supplementary crystallographic information

### Comment

The title compound was obtained as a decomposition product. The organic salt is composed of the pyrrolidinium cation and a chloride anion (Fig. 1). The crystal packing is shown in Fig. 2. In the crystal, both H atoms bonded to N1 of the pyrrolidinium cation are involved in hydrogen bonds with chloride as acceptor. Both can be described according to graph set analysis with a D^1^_1_(2) descriptor on the unitary level. This bonding pattern leads to chains along [1 0 0] which, starting from chloride, can be described according to graph set analysis with a C^2^_1_(4) descriptor on the binary level. The hydrogen bonding pattern is shown in Fig. 3.

The *C_s_* symmetric five-membered pyrrolidinium ring can be described according to Cremer & Pople (1975) by the puckering parameters q_2_ = 0.3061 Å and Φ_2_ = 180.0000. The closest pucker descriptor is an envelope *E*_N1_.

### Experimental

The title compound was obtained as decomposition product of 2,6-bis(pyrrolidinyl)pyridine, which was synthesized according to Folmer-Anderson *et al.* (2005), after 4 months at room temperature.

### Refinement

H atoms were placed in calculated positions (C—H = 0.99 Å, N—H = 0.92 Å) and were included in the refinement in the riding model approximation with *U*_iso_(H) = 1.2 *U*_eq_(C/N).

### Crystal data

**Table:**

C_4_H_10_N^+^·Cl^−^	*F*(000) = 232
*M**_r_* = 107.58	*D*_x_ = 1.146 Mg m^−^^3^
Orthorhombic, *P**n**m**a*	Mo *K*α radiation, λ = 0.71073 Å
Hall symbol: -P 2ac 2n	Cell parameters from 2321 reflections
*a* = 7.4429 (4) Å	θ = 3.1–27.5°
*b* = 9.4104 (5) Å	µ = 0.48 mm^−^^1^
*c* = 8.9021 (4) Å	*T* = 200 K
*V* = 623.51 (5) Å^3^	Block, colourless
*Z* = 4	0.22 × 0.13 × 0.12 mm

### Data collection

**Table:**

Nonius KappaCCD diffractometer	608 reflections with *I* > 2σ(*I*)
Radiation source: rotating anode	*R*_int_ = 0.037
MONTEL, graded multilayered X-ray optics	θ_max_ = 27.5°, θ_min_ = 3.2°
φ and ω scans	*h* = −8→9
4239 measured reflections	*k* = −12→12
756 independent reflections	*l* = −11→10

### Refinement

**Table:**

Refinement on *F*^2^	Primary atom site location: structure-invariant direct methods
Least-squares matrix: full	Secondary atom site location: difference Fourier map
*R*[*F*^2^ > 2σ(*F*^2^)] = 0.040	Hydrogen site location: inferred from neighbouring sites
*wR*(*F*^2^) = 0.120	H-atom parameters constrained
*S* = 1.07	*w* = 1/[σ^2^(*F*_o_^2^) + (0.0625*P*)^2^ + 0.1854*P*] where *P* = (*F*_o_^2^ + 2*F*_c_^2^)/3
756 reflections	(Δ/σ)_max_ < 0.001
31 parameters	Δρ_max_ = 0.21 e Å^−^^3^
0 restraints	Δρ_min_ = −0.23 e Å^−^^3^

### Special details

**Table:**

Refinement. Hydrogen atoms were placed in calculated positions (C–H 0.99 Å for methylene C atoms and N–H 0.92 Å for N atoms) and were included in the refinement in the riding model approximation with U(H) set to 1.2 *U*_eq_(C) for C atoms and 1.2 *U*_eq_(N) for N atoms.

### Fractional atomic coordinates and isotropic or equivalent isotropic displacement parameters (Å^2^)

**Table:**

	*x*	*y*	*z*	*U*_iso_*/*U*_eq_	
N1	0.2072 (3)	0.2500	0.3955 (3)	0.0473 (6)	
H101	0.2341	0.2500	0.2946	0.057*	
H102	0.0843	0.2500	0.4065	0.057*	
C1	0.3204 (4)	0.1712 (3)	0.6283 (3)	0.0781 (8)	
H11	0.2249	0.1348	0.6954	0.094*	
H12	0.4376	0.1348	0.6641	0.094*	
C2	0.2871 (3)	0.1242 (2)	0.4702 (3)	0.0623 (6)	
H21	0.4009	0.0963	0.4208	0.075*	
H22	0.2032	0.0427	0.4677	0.075*	
Cl1	0.29515 (8)	0.2500	0.05610 (7)	0.0488 (3)	

### Atomic displacement parameters (Å^2^)

**Table:**

	*U*^11^	*U*^22^	*U*^33^	*U*^12^	*U*^13^	*U*^23^
N1	0.0466 (12)	0.0500 (13)	0.0452 (12)	0.000	−0.0007 (10)	0.000
C1	0.102 (2)	0.0775 (16)	0.0553 (14)	0.0109 (14)	−0.0060 (13)	0.0106 (12)
C2	0.0803 (16)	0.0411 (11)	0.0657 (14)	0.0045 (10)	0.0013 (11)	0.0050 (9)
Cl1	0.0477 (4)	0.0528 (4)	0.0459 (4)	0.000	−0.0020 (3)	0.000

### Geometric parameters (Å, °)

**Table:**

N1—C2^i^	1.482 (3)	C1—C2	1.495 (4)
N1—C2	1.482 (2)	C1—H11	0.9900
N1—H101	0.9200	C1—H12	0.9900
N1—H102	0.9200	C2—H21	0.9900
C1—C1^i^	1.482 (5)	C2—H22	0.9900
			
C2^i^—N1—C2	105.9 (2)	C1^i^—C1—H12	110.3
C2^i^—N1—H101	110.5	C2—C1—H12	110.3
C2—N1—H101	110.5	H11—C1—H12	108.5
C2^i^—N1—H102	110.5	N1—C2—C1	104.64 (19)
C2—N1—H102	110.5	N1—C2—H21	110.8
H101—N1—H102	108.7	C1—C2—H21	110.8
C1^i^—C1—C2	107.20 (13)	N1—C2—H22	110.8
C1^i^—C1—H11	110.3	C1—C2—H22	110.8
C2—C1—H11	110.3	H21—C2—H22	108.9
			
C2^i^—N1—C2—C1	31.4 (3)	C1^i^—C1—C2—N1	−19.16 (18)

Symmetry codes: (i) *x*, −*y*+1/2, *z*.

### Hydrogen-bond geometry (Å, °)

**Table:**

*D*—H···*A*	*D*—H	H···*A*	*D*···*A*	*D*—H···*A*
N1—H101···Cl1	0.92	2.17	3.091 (3)	180
N1—H102···Cl1^ii^	0.92	2.18	3.097 (2)	177

Symmetry codes: (ii) *x*−1/2, *y*, −*z*+1/2.
